# A spectrally tunable all-graphene-based flexible field-effect light-emitting device

**DOI:** 10.1038/ncomms8767

**Published:** 2015-07-16

**Authors:** Xiaomu Wang, He Tian, Mohammad Ali Mohammad, Cheng Li, Can Wu, Yi Yang, Tian-Ling Ren

**Affiliations:** 1 Institute of Microelectronics, Tsinghua University, Beijing 100084, China.; 2 Tsinghua National Laboratory for Information Science and Technology (TNList), Tsinghua University, Beijing 100084, China.

## Abstract

The continuous tuning of the emission spectrum of a single light-emitting diode (LED) by an external electrical bias is of great technological significance as a crucial property in high-quality displays, yet this capability has not been demonstrated in existing LEDs. Graphene, a tunable optical platform, is a promising medium to achieve this goal. Here we demonstrate a bright spectrally tunable electroluminescence from blue (∼450 nm) to red (∼750 nm) at the graphene oxide/reduced-graphene oxide interface. We explain the electroluminescence results from the recombination of Poole–Frenkel emission ionized electrons at the localized energy levels arising from semi-reduced graphene oxide, and holes from the top of the π band. Tuning of the emission wavelength is achieved by gate modulation of the participating localized energy levels. Our demonstration of current-driven tunable LEDs not only represents a method for emission wavelength tuning but also may find applications in high-quality displays.

## Abstract (web summary)

Wavelength tuning of a light emitting diode (LED) by an external electrical bias would benefit display technologies. Here, Wang *et al*. demonstrate wavelength-tuning from blue to the near-infrared in an all-graphene-based field effect LED by gate modulation

## Graphical abstract

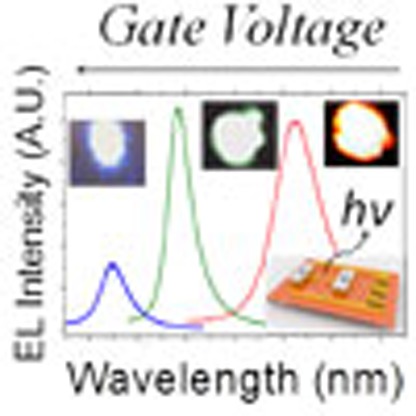

The solid-state light-emitting diode (LED) is a key component of today's semiconductor industry^1^. The successful development of LEDs has enabled plenty of applications such as high-performance communication, low-cost lighting and smart displays. In modern LED industries, controlling the colour of LEDs is a challenging task^2^,^3^,^4^,^5^,^6^. Traditional LEDs are available in different predefined colours whose emission wavelength is adjusted by complex material design and bandgap engineering. To date, the *in situ* controlling of colour in a single device has never been realized, although it is highly desired. Graphene and its derivative material-based photonic devices are currently the focus of intense study^7^,^8^,^9^,^10^,^11^,^12^. Graphene is an amendable platform whose electronic and optical properties can be tailored by chemical and electrical means. This property is particularly interesting as it potentially provides a way to *in situ* control the colour of LEDs. Although various high-performance graphene-based photonic devices have been reported^13^,^14^,^15^,^16^,^17^, unfortunately the development of graphene-based LEDs has been unsuccessful owing to its vanishing bandgap.

A plausible strategy for realizing electroluminescence (EL) may be by creating discrete energy levels by utilizing functional groups, quantum confinement effect or other mid gap states. Unfortunately, the insulating nature of the photoluminescent graphene derivatives, such as functionalized graphene or solution-based graphene quantum dots, negatively affects the carrier injection and disables EL. In summary, a method to fabricate a graphene-based device with a non-vanishing bandgap and charge injection capability is still lacking.

In this article, we demonstrate a desirable combination of a bandgap structure and a bipolar carrier injection in a special type of semi-reduced graphene oxide (GO). Our device formed on a laser-scribed GO surface consists of a series of rGO nanoclusters with many different sizes, which can selectively stimulate a single-colour luminescence by controlling its doping level. The semi-reduced GO network is with a mobility approaches 10 cm^2^ V^−1^ s^−1^, which supports the carrier injection. We report the observation of EL in these semi-reduced GO-based devices. The light-emission spectrum in our device is *in situ* adjusted from blue (*λ*∼450 nm) to red (*λ*∼750 nm) by electrical gating or conditioning the environmental doping. The device shows a high brightness of up to 6,000 cd m^−2^, with efficiency around 1%.

## Results

### Fabrication and characterization of field-effect LED

**Figure 1:**
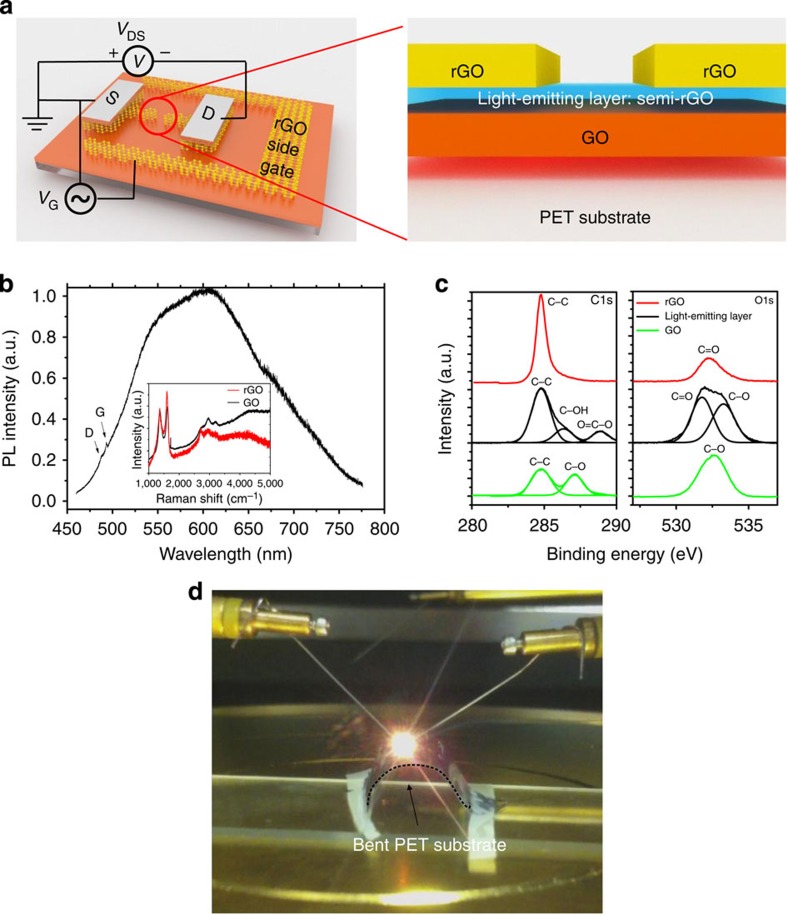
GFLED and characterization. (**a**) Schematic of the GFLED. A distinct semi-reduced GO (blue) at the interface between GO (orange) and rGO (gold) is responsible for light emission. (**b**) PL spectrum of light-emitting layer. The D and G Raman peaks are marked. Inset: Raman spectra of the GO and rGO samples. The GO and rGO samples do not show any PL signals. (**c**) X-ray photoemission spectroscopy spectra of rGO (red), GO (green) and the light-emission layer (black). Spectra from the C 1s and O 1s orbitals are shown. (**d**) Bright red light emission from the GFLED on a flexible polyethylene terephthalate (PET) substrate under a 12 V bias voltage and a 0.1 A drive current. The GFLED size is around 100 × 100 μm. The edge of the bent PET is marked by a dashed line. The bending radius is ∼8 mm.

Figure 1a schematically shows the structure of our all-graphene-based field-effect LED (GFLED). A planar side gate graphene field-effect transistor device was first prepared based on a laser-scribing method (see Methods for details)^18^. The light-emitting layer, which is the interface between the GO and rGO, was then uncovered by using current annealing to remove the highly conductive rGO channel. The typical length of the light-emitting region is 80–120 μm and is located in the centre of the narrow graphene field-effect transistor channel. This central location is determined by the geometric design as the Joule heating rapidly affects the narrowest part of the structure. It is worth mentioning here that the fabrication process was carried out in ambient conditions and does not require any high-vacuum environment, hazardous solution or high-temperature treatment.

The exposed interfacial layer obtained by this method presents three major differences as compared with GO or rGO. First, this interfacial layer is ultra-flat. The RMS thickness obtained from atomic force microscope is several orders smaller than that of GO or rGO. Second, the interfacial layer is colourful, this is in stark contrast to the white colour of GO and the black colour of rGO. Third, and perhaps most importantly, a strong and broadband photoluminescence is solely observed in the interfacial layer, as shown in Fig. 1b. (See Supplementary Figs 1–4 and Supplementary Note 1 for detailed comparison between the interfacial layer and bulk GO/rGO.) The photo-luminescence (PL) spectra of the GO/rGO interfacial layer were obtained by exciting the samples with a continuous wave 457 nm laser with low excitation power (∼100 μW). The interfacial layer showed very broad PL, ranging from about 470 to more than 720 nm (with ∼0.8 eV full-width at half-maximum).

Generally speaking, the zero-bandgap nature of rGO results in a large non-radiative decay rate of stimulated electron–hole pairs, while the energy gap between the π and π* bands of GO is extremely large. All these factors imply that luminescence in rGO and GO should be impossible. The experimentally observed broad PL feature is a strong signature of the presence of localized states, similar as those in rGO quantum dots^19^,^20^. These localized states can be understood by the formation of a transition state during the reduction process: there is a distinct partially reduced GO layer between the GO precursor and the thermally induced rGO as a result of the laser dosage gradient from the surface downwards.

We performed X-ray photoemission spectroscopy to further characterize the chemical composition of the light-emitting layer. A collection of X-ray photoemission spectroscopy spectra is shown in Fig. 1c. Fundamentally, the light-emitting layer can be identified as an intermediate state between GO and rGO in terms of oxygen concentration. The C/O ratio for rGO, the interface layer and GO is 2.10, 0.77 and 0.57, respectively. The chemical states of carbon and oxygen in different layers also show pronounced variation. The C 1s spectra of GO and rGO are similar as per the previously reported typical results^21^. For the GO case, in addition to a large carbon peak composed of graphitic *sp*^2^ and *sp*^3^ C–C bonds, another C–O clear peak can be observed due to the hydroxyls and epoxies. For the rGO case, the spectrum is dominated by the graphitic *sp*^2^ component and the C–O bonds only show a very minute component. However, the interfacial layer exhibits distinct features as compared with the GO and rGO. In addition to an intermediate carbon–carbon bond state, the interfacial layer is also rich in C=O bonds. These bonds are usually attributed to carbonyls. The presence of carbonyls in the GO basal plane signifies incomplete reduction^21^. A similar feature of the transition state was also observed in the O 1s spectra. While rGO and GO are dominated by C=O and C–O peaks, respectively, the light-emitting layer is a mixture of C–O and C=O bonds. The two-component configuration has been used to characterize the transition state in the evolution of reduction of GO^22^.

As described above, the interfacial layer is a special type of partially reduced GO. Generally speaking, the reduction of GO leads to creation of small (2–3 nm) ordered *sp*^2^ clusters isolated within the *sp*^3^ C–O matrix of GO^7^. Therefore, the partially reduced GO likely results in a series of discrete energy levels between π and π* bands by quantum confinement effect^19^. Incident laser stimulates electron–hole pairs in the π band and in these discrete states. Radiative recombination of the electron pairs thus gives rise to a PL effect (and also leads to EL, see discussion below). In this case, the degree of reduction of the rGO determines the features of the PL spectrum, similar to how porous silicon and colloidal quantum dot devices affect the spectrum^6^,^23^.

### Spectrally adjustable EL

**Figure 2:**
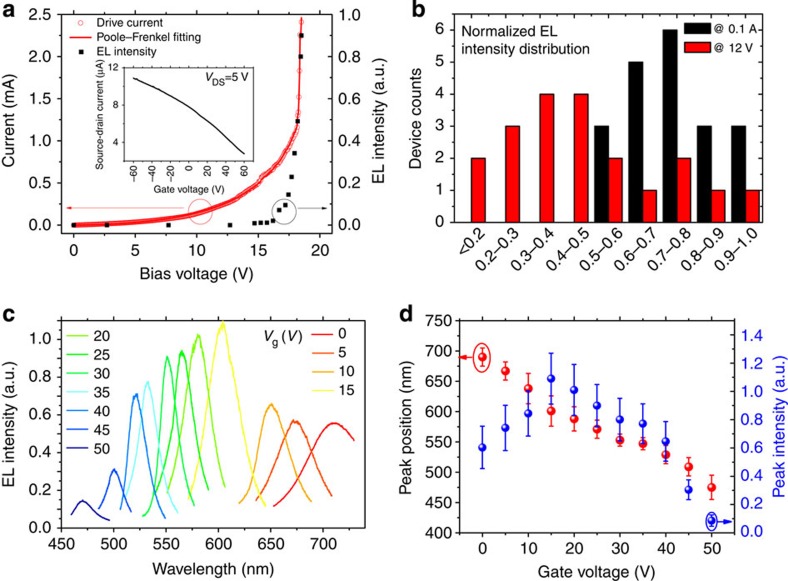
Electroluminescence of GFLED. (**a**) Source-drain current (circles) and EL intensity (solid squares) versus source-drain bias voltage. The device used has a W/L ratio around 0.1 mm/0.1 mm. The solid line shows the Poole–Frenkel fitting of the drive current. The EL peak is at 690 nm. Inset: source-drain current versus gate voltage of the same device, showing a p-type field effect. (**b**) Histogram of EL intensity distribution under fixed voltage and current bias. The data have been obtained from 20 devices. (**c**) Typical EL spectra of a single GFLED. Gate biases are from 0 to 50 V. (**d**) Peak position (wavelength) and intensity of the GFLED EL versus gate voltage. The data have been collected from 19 devices. Error bars (standard uncertainty) are estimated from the device variations.

Once the energy levels with bandgaps are created, EL can be expected if bipolar carriers are injected into the device. Previously reported ‘gapped graphene' (such as GO and graphene quantum dots) is either an insulator or a liquid-phase material, which impair the carrier injection. Fortunately, the interfacial layer (although also a type of GO), is a p-type semiconductor with a good conductivity (Fig. 2a inset; also see Supplementary Figs 5–7 and Supplementary Note 2 for characterization of its field effect). In addition, an enhanced carrier injection was also identified under a strong bias field, manifested by the rapidly increasing current versus bias voltage in Fig. 2a. Accompanied by the pronounced free carrier injection process in the device, we observed EL along the interfacial layer. The emitted light is bright and easily identified by the naked eye (see Fig. 1d). We fabricated 20 devices to characterize the bias-dependent EL intensity. Typical relative emission intensity versus different drain bias and the EL intensity distribution are plotted in Fig. 2a,b, respectively. Notably, the EL intensity is controlled by drive current. As shown in Fig. 2b, the devices are relatively uniform under a fixed 0.1 A current bias (*I*_max_/*I*_min_<2). In contrast, the relative EL intensity presents large device to device variation (*I*_max_/*I*_min_∼10) under a fixed 12 V voltage.

An exceptional observation in this work is that the colour of EL emission can be continuously tuned from light blue to dark red by adjusting the Fermi levels. The Fermi level and the doping level of the GFLED can be modulated either electrically or chemically. In this paper, we use a side gate field to control the Fermi level; however, the colour also changes in response to ambient doping, enabling sensing applications. Typical emission images, spectra recorded at different side gate voltages during the EL measurement and spatially resolved EL are shown in Figs 1d and 2c and Supplementary Fig. 8. The EL peaks exhibit sharp single Lorentzian shapes. As shown in Fig. 2c, the peak emission wavelength shifts from 690 nm at zero gate voltage to 470 nm under 50 V gate voltage. On one hand, the EL peaks present similar narrow full-width at half-maximum (∼0.2 eV), whereas on the other hand, the relative intensity of the EL is found to vary under different bias voltages. For instance, the maximum luminance and the luminous efficacy of the blue light are much lower than the red light. Approximately 4.8 lm W^−1^ (corresponding to ∼0.7% external quantum efficiency, also see Supplementary Figs 9 and 10 for details) and 6,000 cd m^−2^ red and green lights were measured under a 12 V bias and a 0.1 A drive current, whereas 0.67 lm W^−1^ (corresponding to ∼0.1% external quantum efficiency) and 800 cd m^−2^ blue light was measured under a 16.5 V bias and a 0.1 A drive current. Overall, the broad PL can be roughly regarded as the envelope of all these possible EL spectra. This strongly suggests that the EL results from a selective excitation of inhomogeneous luminescence of semi-reduced GO, as observed in previously reported rGO quantum dots. A possible explanation of the field effect-modified EL will be discussed in the following part.

Currently, the efficiency of GFLEDs is relatively low. We hypothesize that two factors limit the efficiency. First, as shown in Supplementary Fig. 9, the efficiency tends to saturate at a large current density. We attribute this saturation to the inefficient injection of electrons caused by heat dissipation, that is, much of the supplied power is lost to heat dissipation. In other words, the saturation is caused by the inefficient injection of electrons (discussed below). The large amount of heat dissipated can also break the devices rapidly. The emission lifetime of these devices are several tens of seconds in ambient conditions. This short lifetime can be attributed to vigorous oxidation in air. However, these devices have a much longer half-life in vacuum and tests have revealed continuous and stable emission for up to 2 h, suggesting protective coatings may help to avoid structural damage in practical devices. Second, in the absence of an intersystem cross path, only singlet excitons can be harvested, which suggests that the efficiency upper limit of GFLED is 25%. Therefore, the promotion of efficiency requires improved carrier injection and introducing intersystem crossing in future works.

### EL mechanism

Next, we proceed to discuss the possible EL mechanism. The simultaneously rapid increase of the current and EL intensity clearly indicates that carrier injection plays a central role in the EL process. Owing to the p-type nature of the light-emitting layer, electron injection is the key factor of understanding the EL process. At the current state, the exact mechanism of electron injection is not well known. On the basis of the various evidences, we hypothesize that the electric field-assisted thermal ionization, also known as Poole–Frenkel emission, is the most probable mechanism.

**Figure 3:**
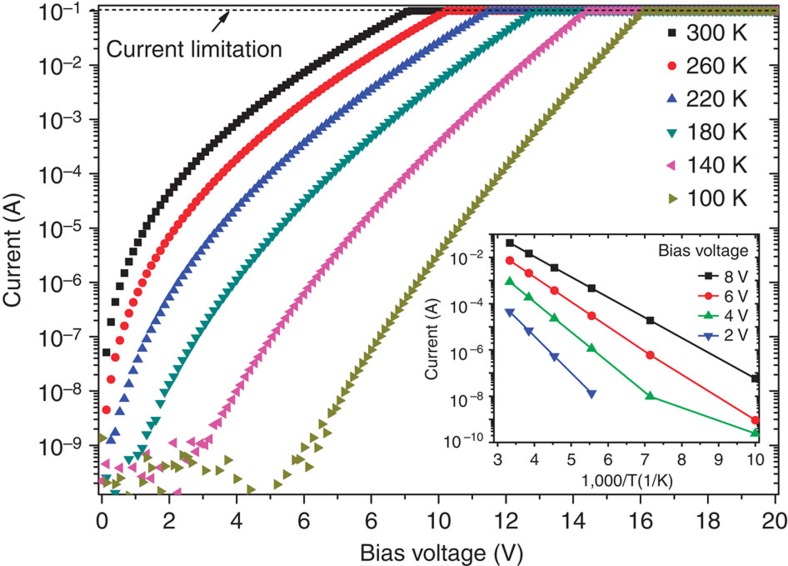
GFLED two-terminal temperature-dependent transport. Source-drain current versus voltage bias at different temperatures. The current is limited to 0.1 A to avoid device breakdown. Inset: Arrhenius plot of source-drain current at bias voltages of 2, 4, 6 and 8 V.

**Figure 4:**
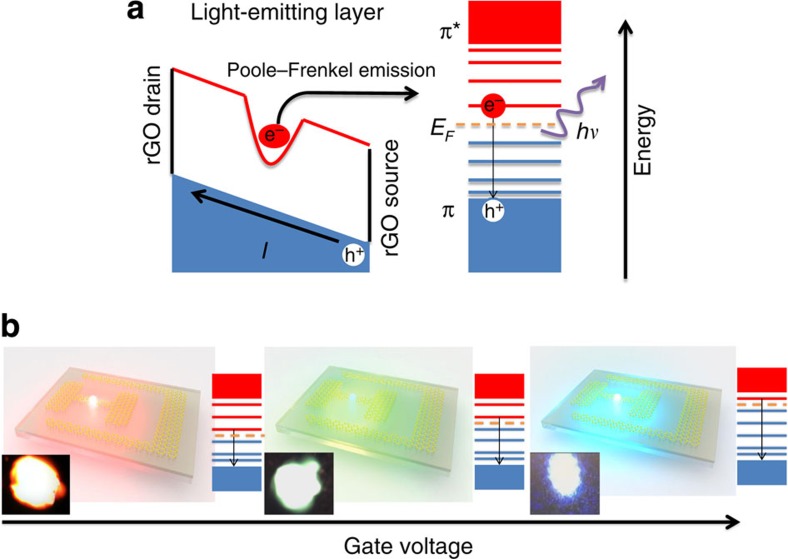
GFLED light-emission mechanism. (**a**) A schematic of the charge injection process. The trapped electrons are excited to the lowest unoccupied discrete energy level by Poole–Frenkel emission. And the excited electron recombines with the hole in the π band, resulting in photon emission. (**b**) Schematic of the gate voltage-dependent EL. The Fermi level determines the lowest unoccupied energy state that mainly participates in the radiative recombination. Inset: corresponding emission images from a real device.

We performed temperature-dependent electrical measurements to investigate the electron injection process. Figure 4a shows the Poole–Frenkel plot of *I*–*V* characteristics of a typical device. As illustrated in the inset of Fig. 3, a linear relationship fits well to the measured conductivity in the Arrhenius plot, indicating the electrons inject into the system on a charge emission manner. The highly linear feature in the log(*I*) versus *V*^1/2^ plot signifies the *I*–*V* curves agree well with the Poole–Frenkel current law (see Supplementary Note 3 for details)^24^ The Poole–Frenkel effect is usually understood as a means by which trapped electrons get out of Coulomb binding and move to the conduction band. Figure 4a schematically shows how this effect applies to the GFLED. The oxide-rich light-emitting layer has a high density of electron trap states. Under high field, the localized electrons are excited into the lowest unoccupied discrete energy level. Radiative recombination between the thermally emitted electrons and the free holes results in the EL.

This mechanism also explains the observed spectral shift, as shown in Fig. 4b. Gating graphene lifts up the chemical potential and thus the energy level of the lowest unoccupied discrete state; therefore, the excited electron energy is increased. Through this way, the EL peak can be adjusted within the whole PL range; and the relative emission intensity is determined by the distribution of density of states of the discrete energy levels.

Several mechanisms, such as thermal emission^25^, Fowler–Nordheim tunnelling^26^,^27^ and impact excitations^28^,^29^,^30^ may also cause the EL in the GFLED. However, none of them can consistently explain all the findings of this work. Here we discuss the validity of these possibilities to GFLEDs. First of all, the featureless electroluminescence peak would correspond to a local temperature above 4,500 K if the EL results from blackbody radiation coming from Joule heating. This would rule out thermal emission as the light-emitting mechanism. Second, Fowler–Nordheim tunnelling, which is a main charge injection mechanism in porous silicon EL device, may also work for the GFLED^26^. Considering the planar device structure, the tunnelling process most likely occurs around the drain electrode that holds the largest band bending. However, this contradicts the light emission observed in the middle of the device. Finally, impact excitation/ionization is another plausible EL mechanism and has been used to successfully describe the EL phenomenon in carbon nanotubes^28^,^29^,^30^. However, the threshold voltage in an impact ionization process is always accompanied by a negative temperature coefficient. Therefore, the observed positive temperature coefficient clearly rules out the impact ionization mechanism.

## Discussion

In summary, we have reported the design and implementation of a LED device based on a special type of semi-reduced GO that forms in the rGO/GO interface. By yielding a series of discrete energy levels between the π–π* gap and injecting free carriers into those states, EL is successfully achieved. The *in situ* tuned wavelength of the EL exemplifies a kind of novel light-emitting device that is highly desired for high-color-quality displays and high-quality lightings. The precise spectral tunability, compact device structure, high performance and straightforward fabrication point to the commercial prospects of such a device. We anticipate our findings to be a prospective point for commercialization of graphene devices. Furthermore, the observance of EL itself addresses the lack of a light source component in graphene-based photonic devices, paving the way for all-graphene integrated photonics. This device opens the possibility of fabricating carbon-based photonic devices due to the henceforth availability of graphene-based LEDs.

## Methods

### Sample preparation

GFLEDs were fabricated based on a side gate field-effect transistor structure. An ∼1 μm thick GO (XFNANO Materials Tech) film was prepared by drop casting on a flexible polyethylene terephthalate substrate. Conductive rGO traces with ∼100 μm width were then patterned by a previously reported laser-scribing method within a DVD drive^31^. Silver paste electrodes were fabricated to improve the contact. In the scribing process, the DVD drive's 788 nm laser (5 mW) was used to gradually reduce the stacked GO films to rGO. After that, the as-prepared devices were annealed by a 10 mA current to expose the interfacial layer.

### Electrical measurement

Electrical measurements were carried out within a Lakeshore CPX-VF-457 cryogenic probe station using a Keithley 4200 semiconductor analyser. Electrical doping were achieved by using a side gate that surrounds the GFLED. The GO acts as a dielectric with a capacitance of 160 nF (see Supplementary Fig. 5 for details).

### PL and EL measurements

The luminescence measurements were performed using a Renishaw inVia micro-Raman spectroscope equipped with 457, 514 and 633 nm lasers. The spot diameter was around 1 μm under a × 100 objective lens. For PL measurements, the laser powers was set at 100 μW to avoid breaking the samples. The typical integrating time was 10 s. For EL measurements, a dual-channel Keithley 2612 source meter supplied the electrical and gate bias.

## Additional information

**How to cite this article:** Wang, X. *et al*. A spectrally tunable all-graphene-based flexible field-effect light-emitting device. *Nat. Commun.* 6:7767 doi: 10.1038/ncomms8767 (2015).

## Supplementary Material

Supplementary Information Supplementary Notes 1-3 and Supplementary Figures 1-10
