# Supplementary material for: A spectrally tunable all-graphene-based flexible field-effect light-emitting device
Source: Nat Commun. 2015 Jul 16;6:7767. doi: 10.1038/ncomms8767 (PMC4518298; doi:10.1038/ncomms8767)
Supplement: Supplementary Information — Supplementary Notes 1-3 and Supplementary Figures 1-10 [file ncomms8767-s1.pdf]

Supplementary Note 1. Comparision bewteen GO, rGO and their interfacial layer

The interfacial layer between GO and rGO presents a totally diferent mophology and chemical properties as compared with both materials. The photograph of the interfacial layer exhibits a color – this is in stark contrast to the white color of GO and the black color of rGO, as shown in supplementary Fig.1.

To further examine the light-emitting interficial layer, we performed nanoscale morphology analyses using AFM.The microscopic morphology present distict features for the interfacial layer (supplementary Fig.2) and GO (supplementary Fig.3). Firstly, the interfical layer has a very small roughness (RMS 0.594nm) in comparison with GO (4.28 nm). Secondly, several tiny and distinct ripples are recorded in the interfacial layer.

Finally, PL measurements were also carried out for GO and rGO, as shown in supplementary Fig.4. As discussed in the main text of the manuscript, both GO and rGO present pronounced Raman signals only. Although a very weak and broad PL spectrum was observed in the rGO samples, it likely arises from the interfacial layer underneath the rGO. We believe this to be the case as the PL spectrum is strikingly similar to what was observed previously from the interfacial layer. It should be noted that due to the strong resonance energy transfer, graphene (and rGO) presents a strong luminescence quenching effect, suggesting the current annealing step is necessary for the rGO QD LEDs.

Supplementary Note 2. Field-effect Characteristics of the GFLEDs

Firstly, we directly measured gating efficiency in our device based on a capacitor configuration which is similar as the Metal-Insulator-Semicondcutor structure. As shown in the inset of the supplementary Fig.5, we fabricated a capacitor by the laser scribing method. The two rGO regions were defined as two capacitor electrodes, which in our device act as the side gate and source electrode respectively. The *C-f* measurement of this structure clearly demonstrates our structure is with a capacitance around 4×10^{-4} F/m^2. For comparison, we also measured the capacitance of 300 nm SiO_2 substrate, which results in a 1.15×10^{-4} F/m^2 capacitance. After that, the electrical and optical characteristics of GFLED under gate bias are illustrated in supplementary Fig. 6.

Supplementary Note 3. Poole-Frenkel Modeling of the *I-V* curve of GFLED

The I-V curve of the GFLED obeys a Poole–Frenkel relationship which describes the conductance of electricity in an electrical insulator. The physical process can be understood within a release of free carriers from deep traps. In the insulator, the electrons are generally trapped in localized states. Injected holes are accelerated by the strong electric field in the insulator and will transfer that electron enough energy to get out of its localized state, and move to the conduction band.

In this process, the source-drain drive current fits well to

$$I \propto E \exp\left(\frac{-q(\Phi_B - \sqrt{qE/\pi\varepsilon})}{k_B T}\right)$$

where q is unit charge, k_B is Boltzmann constant, T is temperature, Φ_B is zero-bias built in potential between the metal and rGO QDs, and ε is electrical permittivity.

The average applied electric field E can be expressed as

$$E = \frac{1}{l}(V - IR_s)$$

in which V is the voltage across the LED, l is the device channel length, and R_s is the parasitic contact resistance.

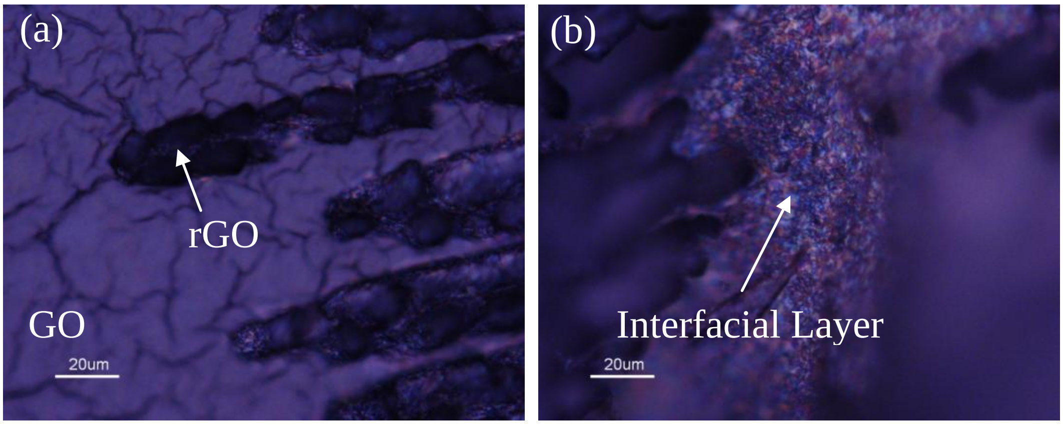

Supplementary Figure 1: Photograph of GO, rGO and GO/rGO interfacial layer. Images of a, rGO on GO prepared by laser scribing, and b, the interfacial layer.

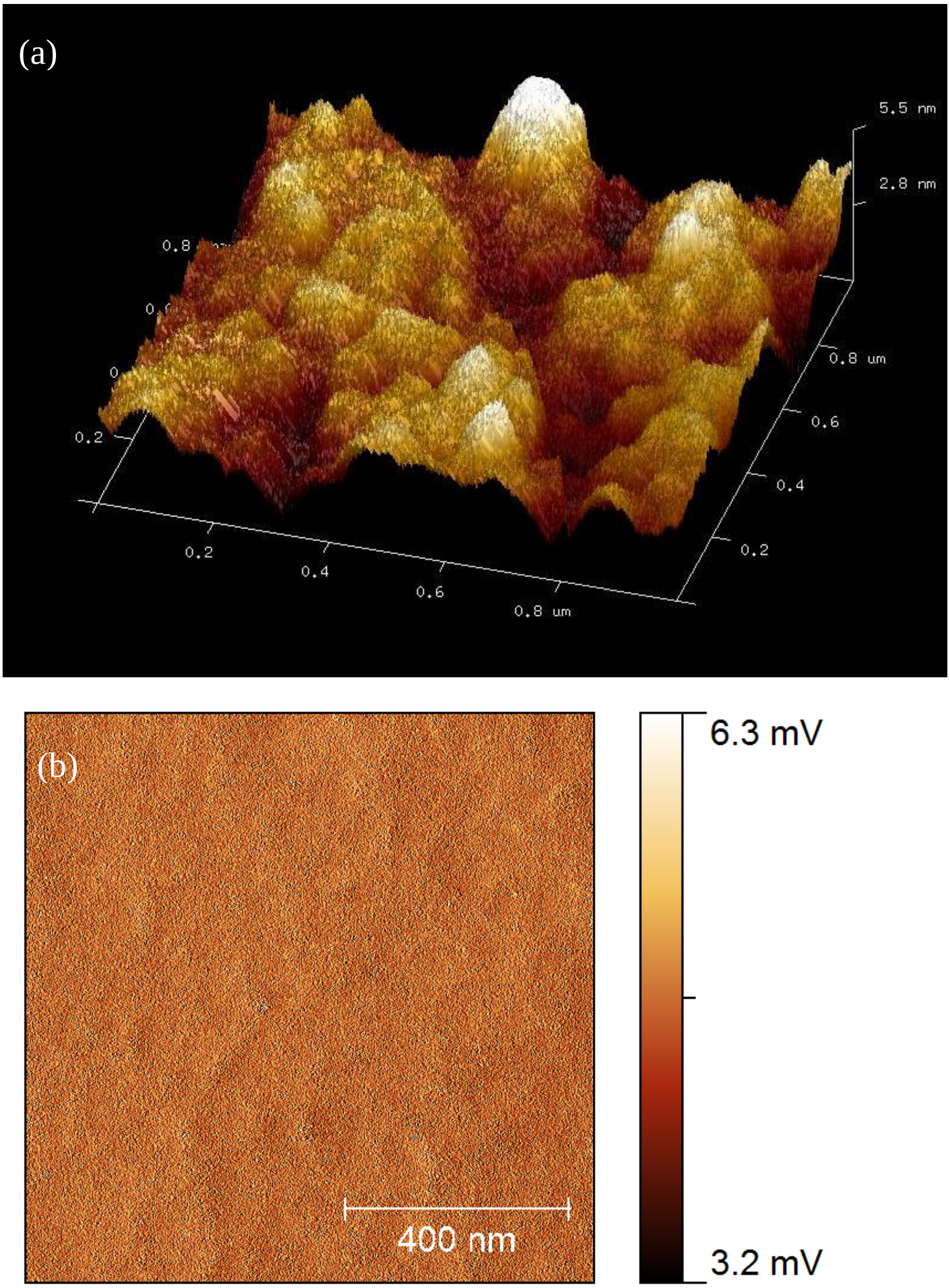

Supplementary Figure 2: AFM images of the GO/rGO interfacial Layer. a. The topography and b. corresponding phase image are both shown.

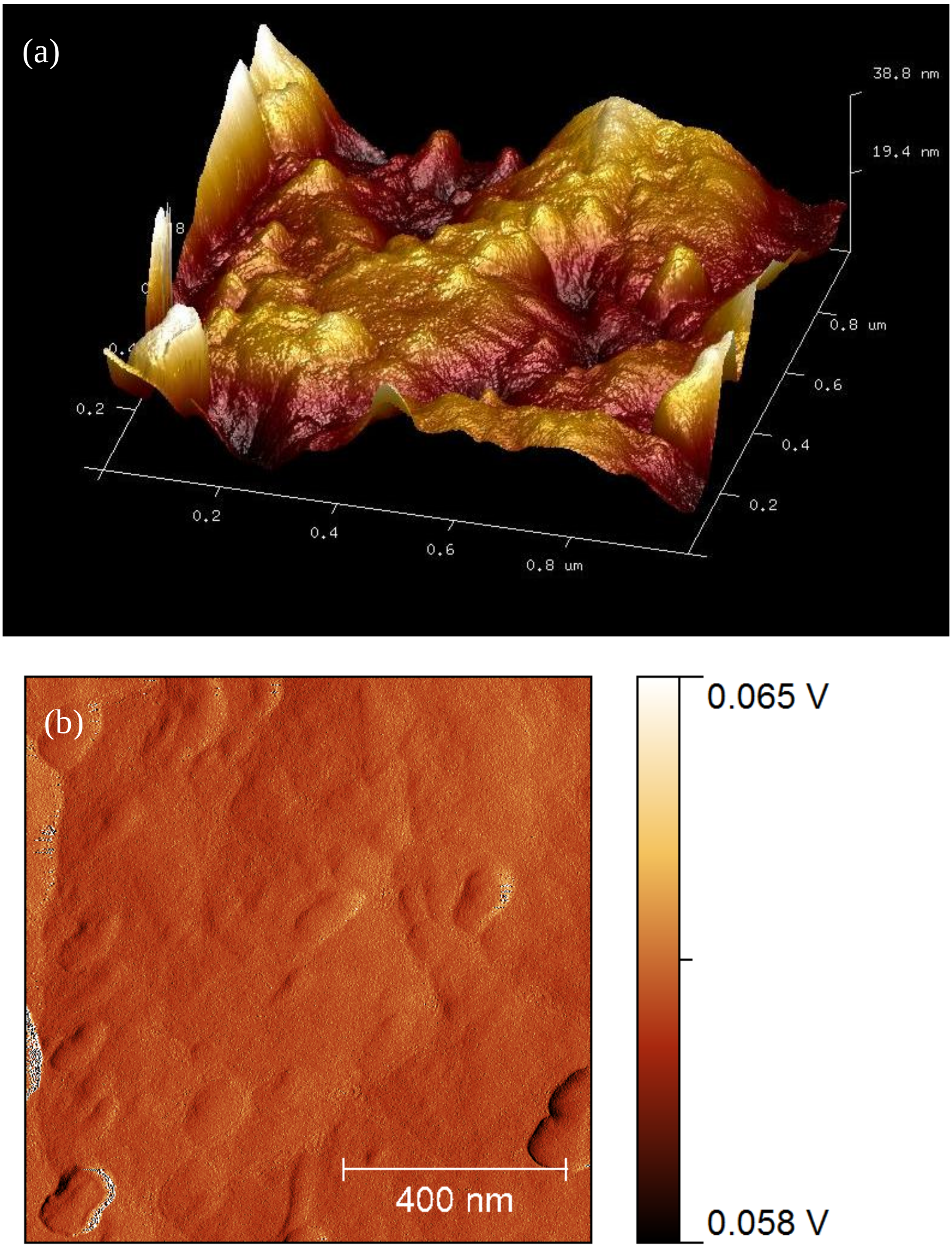

Supplementary Figure 3: AFM images of the GO. a. The topography and b. corresponding phase image are both shown.

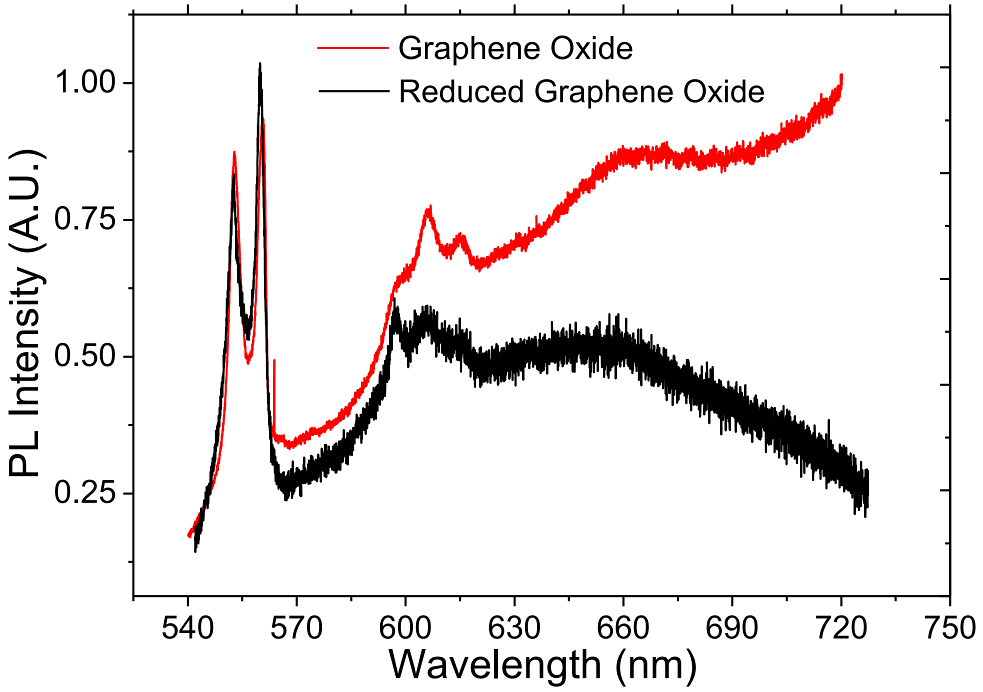

Supplementary Figure 4: PL measurements of GO and rGO

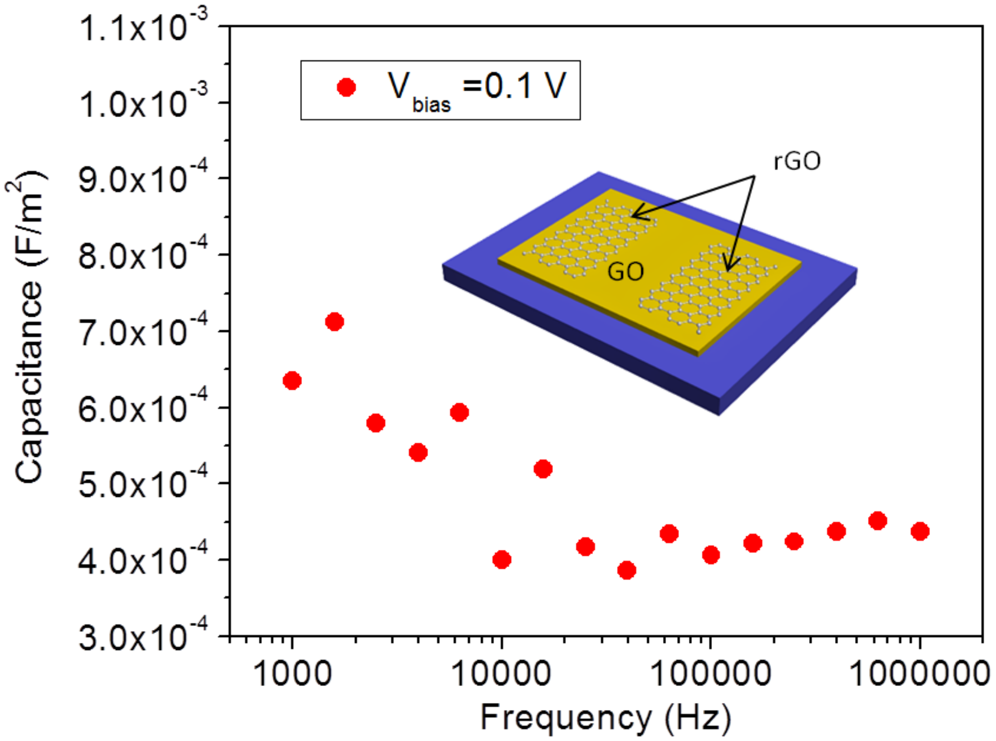

Supplementary Figure 5: Capacitance of GO dielectrics. C-*f* measurements were carried out on a Metal-Insulator-Metal structure. The two rGO regions were defined by laser-scribing as two capacitor electrodes, which in the GFLED act as the side gate and source electrode respectively.

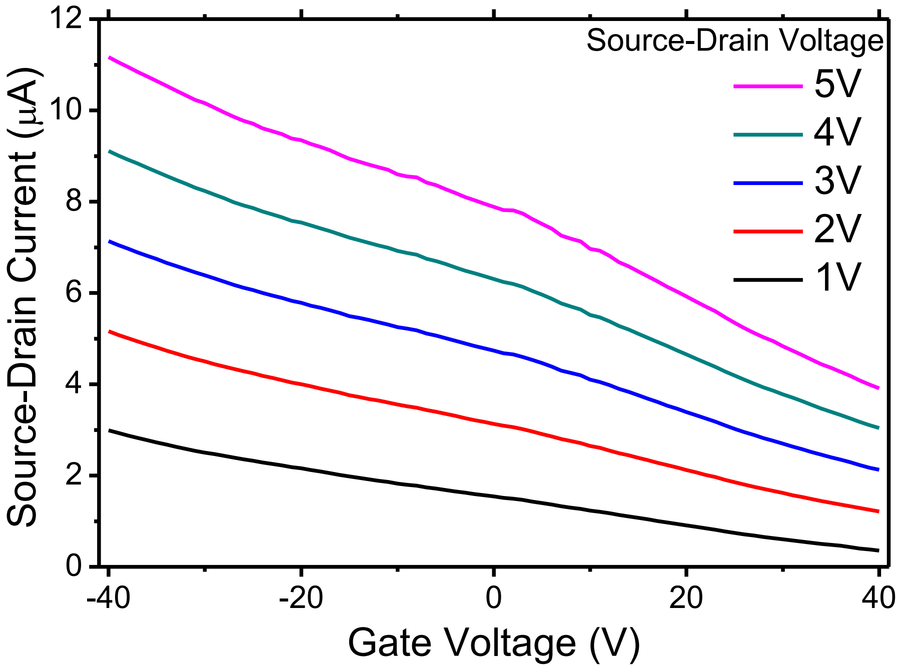

(a)

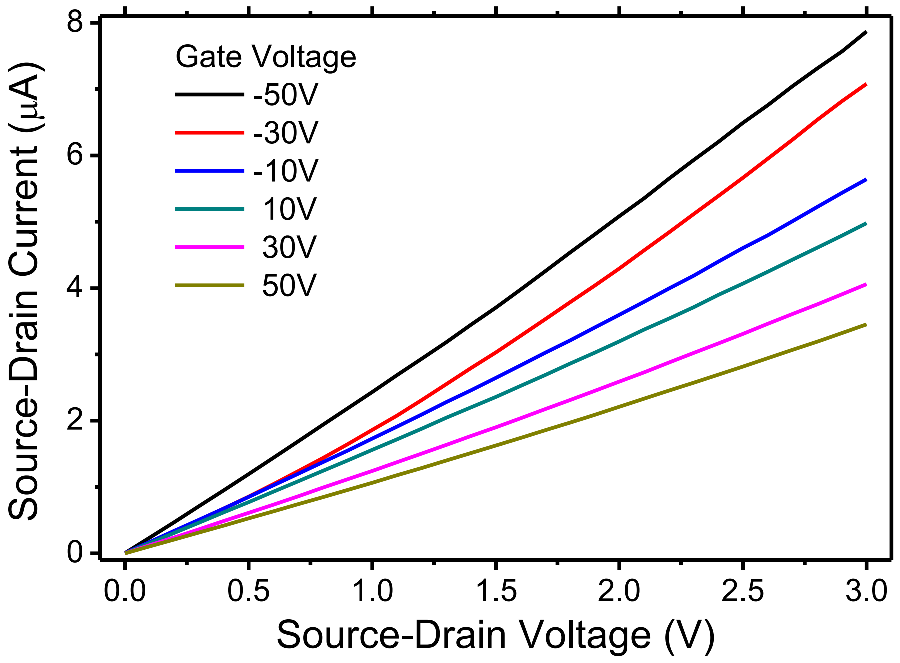

(b)

Supplementary Figure 6: Electrical Charcateristics of GFLED (a) Transfer Curves of a typical GFLED under different source-drain bias (b) Output Curves of a typical GFLED under different source-drain bias

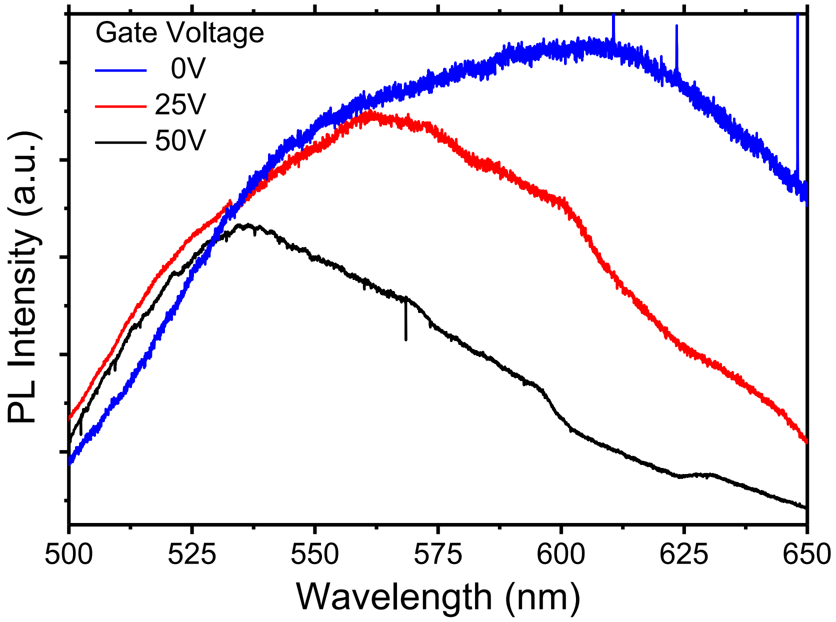

Supplementary Figure 7: Gate-Dependent PL of a typical GFLED

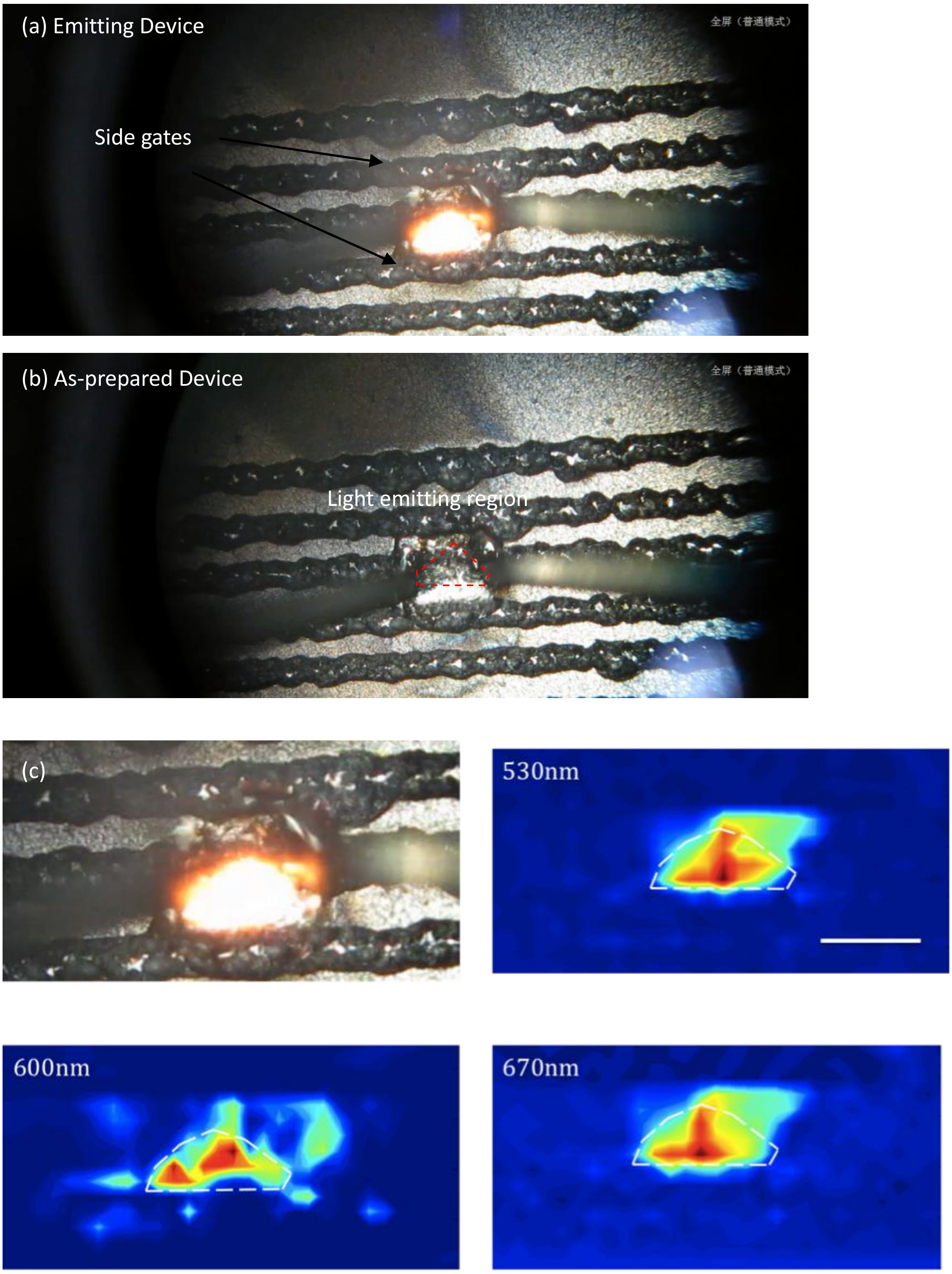

Supplementary Figure 8: Spatial resolution of GFLEDs. Spatially-resolved EL of (a) a working device and (b) the as-prepared device. c. PL mapping at 530, 600 and 670nm illustrate the emission sites of the device. Scale bar: 100 μm

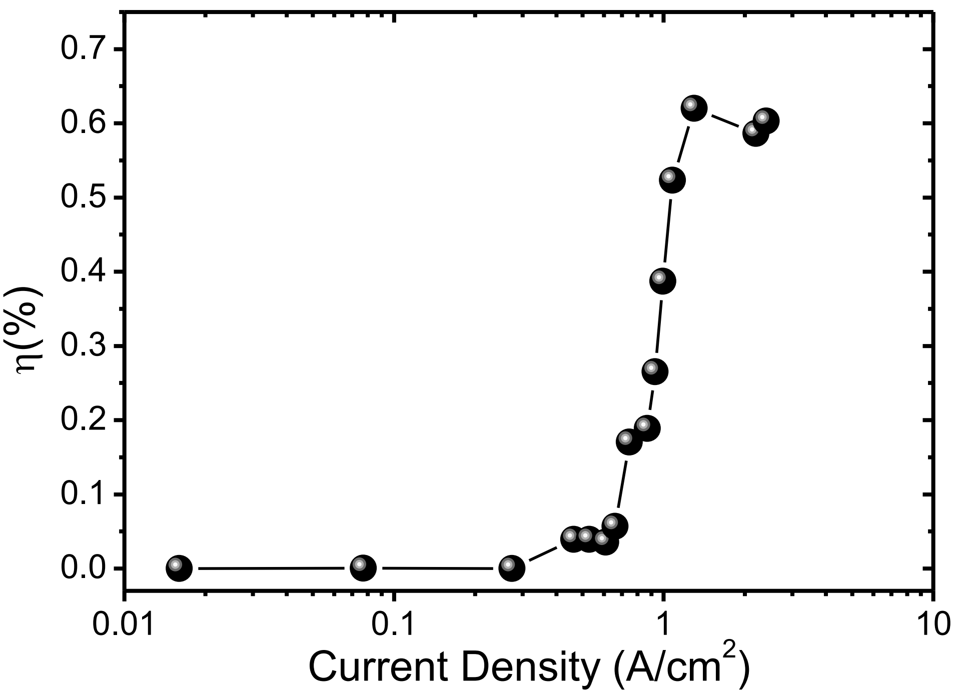

Supplementary Figure 9: Efficiency of a GFLED as a function of Current Density

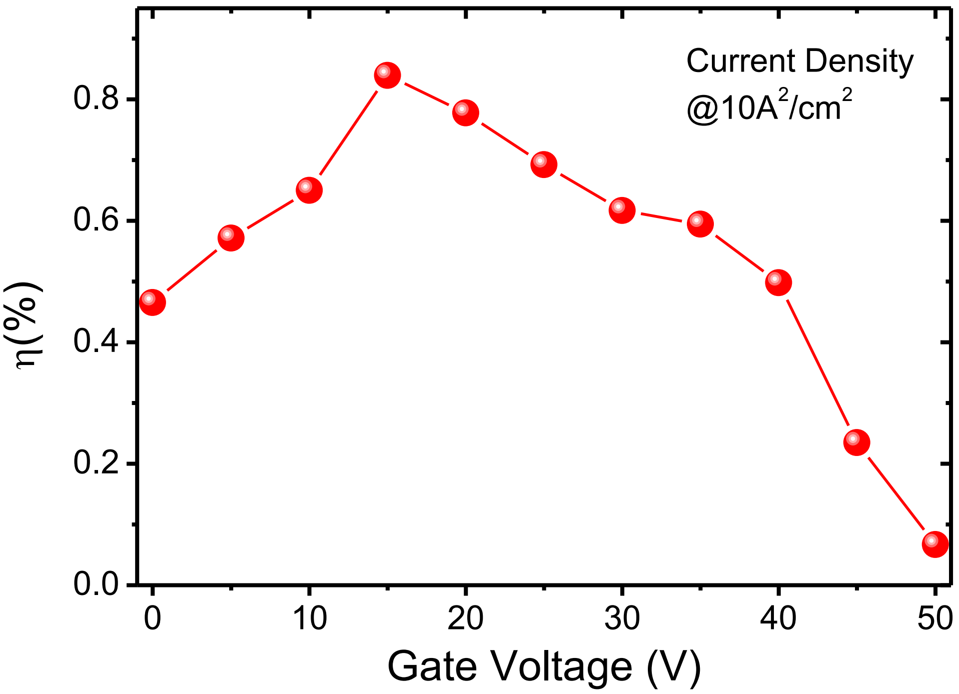

Supplementary Figure 10: Efficiency of a GFLED as a function of gate voltage
